# Plant Rhizosphere Selection of Plasmodiophorid Lineages from Bulk Soil: The Importance of “Hidden” Diversity

**DOI:** 10.3389/fmicb.2018.00168

**Published:** 2018-02-13

**Authors:** David Bass, Christopher van der Gast, Serena Thomson, Sigrid Neuhauser, Sally Hilton, Gary D. Bending

**Affiliations:** ^1^Department of Life Sciences, Natural History Museum, London, United Kingdom; ^2^Centre for Environment, Fisheries and Aquaculture Science, Weymouth, United Kingdom; ^3^School of Healthcare Science, Manchester Metropolitan University, Manchester, United Kingdom; ^4^School of Life Sciences, University of Warwick, Coventry, United Kingdom; ^5^Institute of Microbiology, University of Innsbruck, Innsbruck, Austria

**Keywords:** plasmodiophorid, *Plasmodiophora*, rhizosphere, oilseed rape, wheat, eDNA, soil

## Abstract

Microbial communities closely associated with the rhizosphere can have strong positive and negative impacts on plant health and growth. We used a group-specific amplicon approach to investigate local scale drivers in the diversity and distribution of plasmodiophorids in rhizosphere/root and bulk soil samples from oilseed rape (OSR) and wheat agri-systems. Plasmodiophorids are plant- and stramenopile-associated protists including well known plant pathogens as well as symptomless endobiotic species. We detected 28 plasmodiophorid lineages (OTUs), many of them novel, and showed that plasmodiophorid communities were highly dissimilar and significantly divergent between wheat and OSR rhizospheres and between rhizosphere and bulk soil samples. Bulk soil communities were not significantly different between OSR and wheat systems. Wheat and OSR rhizospheres selected for different plasmodiophorid lineages. An OTU corresponding to *Spongospora nasturtii* was positively selected in the OSR rhizosphere, as were two genetically distinct OTUs. Two novel lineages related to *Sorosphaerula veronicae* were significantly associated with wheat rhizosphere samples, indicating unknown plant-protist relationships. We show that group-targeted eDNA approaches to microbial symbiont-host ecology reveal significant novel diversity and enable inference of differential activity and potential interactions between sequence types, as well as their presence.

## Introduction

Plant roots release considerable amounts of labile exudates and debris into the soil, which results in intense microbial activity in the rhizosphere soil which surrounds roots, and the selection of communities which are structurally and functionally distinct from the bulk soil (Morgan et al., 2005) The rhizosphere microbiome can have major impacts on plant growth and nutrition, which can be both positive and negative, through complex direct and indirect interactions. Although it is widely appreciated that a broad range of microbial groups can inhabit the rhizosphere, most studies have focussed on bacteria and fungi (Mendes et al., 2013; Philippot et al., 2013). For these groups, a range of factors can determine the specific communities which assemble in the rhizosphere, including plant characteristics such as genotype and age, environmental properties including soil type and climate, and in agricultural soils, management interventions such as crop rotation and fertilization (Bennett et al., 2012). Protists are also components of the rhizosphere microbiome (Mendes et al., 2013), and can also have marked impacts on plant growth through direct and indirect pathways (Bonkowski, 2004). However, they are typically not considered in studies of rhizosphere microbiology, largely because culture independent techniques to profile complex protist communities remain limited (Adl et al., 2014), with the result that there is little understanding of the factors which shape protist communities in the rhizosphere.

There is one protist group in particular—plasmodiophorids—that rarely receive attention in rhizosphere ecology studies yet are well known as plant pathogens and virus vectors. Plasmodiophorids (Rhizaria; Endomyxa; class Phytomyxea, Order Plasmodiophorida) are parasites and symbionts of angiosperms and stramenopiles (Bass et al., 2009; Neuhauser et al., 2014). Plasmodiophorid diversity is much greater than the few known parasitic taxa and their broader role(s) in the rhizosphere are of great interest. Plasmodiophorids form obligate associations with their hosts, which are often green plants, but in some instances, they can also infect other parasites including heterotrophic stramenopiles, e.g., *Woronina pythii*, which infects *Pythium* spp. (Dylewski and Miller, 1983; Neuhauser et al., 2014). Plasmodiophorids are the causative agents of economically significant diseases of crops including brassicas, potatoes, and grain crops (e.g., maize, rice, wheat, sorghum). *Plasmodiophora brassicae* is the commercially most important and best studied plasmodiophorid, causing clubroot disease in cruciferous plants such as oilseed rape (OSR). Clubroot has been shown to result in average crop losses of 10-15% on a global scale (Dixon, 2009, 2014; Hwang et al., 2012). Other plasmodiophorids include *Spongospora subterranea*, which causes powdery scab of potato and can also vector Potato Mop Top Virus (Beuch et al., 2015; Falloon et al., 2015). *Polymxa graminis* can infect most graminaceous crops where it does not cause disease symptoms itself, but can transmit several viruses, such as soil-borne wheat mosaic virus (SBWMV), which is considered as one of the most important diseases of winter wheat in the Central and Eastern USA (Kanyuka et al., 2003). *Polymyxa betae* vectors Beet Necrotic Yellow Vein Virus, which causes sugar beet “rhizomania,” resulting in ca. 10% loss or the world production of sugar beet (Lemaire et al., 1988; Desoignies et al., 2014; Hassanzadeh Davarani et al., 2014; Biancardi and Lewellen, 2016).

As obligate biotrophs, plasmodiophorids require specific, living hosts for the completion of their life cycle and to enable them to successfully reproduce. But beyond these “primary” hosts, in which the full life cycle can be completed, some plasmodiophorids are associated with a variety of alternative hosts, in which often only the sporangial part of the life cycle can be completed, which results in the formation of short lived zoospores. For example, *Spongospora subterranea*, whose primary hosts belong to the Solanaceae, can also cause small sporangial infections in hosts within the plant families Poaceae, Brassicaceae, Leguminosae and Geraniaceae (Qu and Christ, 2006). *Polymyxa graminis*, which has primary hosts including most Poaceae, can for example also infect *Arabidopsis thaliana* as alternative host (Smith et al., 2011). Similarly, *Polymyxa betae*, which was considered to be a specialist pathogen of Chenopodiaceae (Desoignies et al., 2014), has now been found to also infect graminaceaous hosts such as wheat (Smith et al., 2013).

The complexity of the plasmodiophorid life cycle (Bulman and Neuhauser, 2017), coupled with their small size (3–6 μm), makes them difficult to study. Specimen-independent molecular probing and sequencing of microbial diversity in environmental samples, referred to as eDNA (environmental DNA) offers an alternative perspective on elusive and cryptic microbes to more classical organism-centric studies (Bass et al., 2015). A recent eDNA study (Neuhauser et al., 2014) investigated plasmodiophorid biodiversity by analysing root and soil-associated plasmodiophorids in a range of habitats, including a vineyard, flood plain and glacier forefield. Eighty-one new OTUs were discovered from just 6 locations, significantly adding to the 41 known phytomyxid (combined plasmodiophorid and phagomyxid) lineages. This suggests that many lineages remain uncharacterized, the biological function of which is unknown. Given the importance of plasmodiophorids as crop disease agents and viral disease vectors, understanding the recently demonstrated “expanded” diversity and distribution of the group within agricultural systems is important to more fully understand crop health, parasite load, and organismal interactions.

A novel insight into the structure of plasmodiophorid communities and the factors modulating this can be deduced by objectively partitioning the community into core and satellite taxa (van der Gast et al., 2011). By decomposing the relationship between distribution (number of sample communities that taxa occupy) and mean abundance (across those sample communities), core members of the plasmodiophorid communities can be identified as those that are locally abundant and non-randomly distributed. With the rare satellite taxa defined as those that are typically in low abundance and randomly distributed through sample communities (Hanski, 1982; Magurran and Henderson, 2003). This is particularly relevant given that so little is known about the ecological processes acting on plasmodiophorid communities, including immigration and extinction, and competition and niche partitioning, all of which are currently unknown in plasmodiophorids (Ulrich and Zalewski, 2006).

For this study, we designed plasmodophorid-specific PCR primers to generate 18S rDNA amplicons suitable for targeted amplicon high-throughput sequencing (HTS), to determine the local scale drivers of plasmodiophorid distribution and to compare their community assembly in rhizosphere/root and bulk soil samples. The use of HTS approaches for functional ecology studies of micro-eukaryotes lags behind that for diversity and/or phylogenetically oriented diversity. However, the use of HTS for both types of study confers the advantage of being able to detect a phylogenetically defined set of lineages without biases associated with sampling for, and accurately identifying, small and cryptically differentiated microbes. We demonstrate that HTS methods can provide information about host associations of microbial lineages without prior knowledge of the microbes involved or any assumption of specific host-microbe relationships.

## Materials and methods

### Experimental design and sampling

A field trial designed to investigate the influence of OSR cultivation frequency on crop yield (Hilton et al., 2013) was used to investigate the roles of crop species (wheat and OSR), sampling time and OSR rotation frequency for controlling plasmodiophorid community assembly in rhizosphere and bulk soil compartments. The field trial was in East Anglia, UK (52° 33′ N and 1° 2′ E), on a sandy clay loam soil with a pH of 6.6 and available P, K, Mg, and SO_4_ of 32.4, 111, 28, 30.6 mg kg ^−1^ respectively. In the trial, OSR (cv. Winner) and winter wheat (cv. Brompton) were grown together in different rotation frequencies over a 5 year period (Table 1). The trial was designed so that each rotation was available for sampling (in different plots) in the 2007 and 2008 harvest season. The field was ploughed and pressed each season before establishment. Drilling occurred at the beginning of September for OSR, mid-September for the first winter wheat, followed by mid-October for subsequent wheat. Local commercial best practice was adhered to for pesticide and fertilizer inputs. For OSR this included autumn herbicide (diflufenican) and insecticide (cypermethrin), and spring insecticides (lamda cyalothrin and cyclohexadione), together with nitrogen and sulfur inputs of 200 and 30 kg ha^−1^ respectively. For wheat this included autumn herbicide (diflufenican) and spring fungicides (propiconazole, chlorothalnil and cyproconazole) and 100 kg N ha^−1^.

**Table 1:** Similarity of percentages (SIMPER) analysis of community dissimilarity (Bray-Curtis) between plasmodiophorid metacommunities from each habitat.

	**Wheat Rhizosphere**		**Wheat Soil**		**Oil seed rape Rhizosphere**		**Oil seed rape Soil**				
	**Occupancy**	**Abundance**	**Occupancy**	**Abundance**	**Occupancy**	**Abundance**	**Occupancy**	**Abundance**	**Av. dissim**	**Contrib. %**	**Cumulative %**
OTU 1	96.4	9.6	100	24.60	100	76.40	100	32.1	21.58	32.68	32.68
OTU 24	78.6	48.3	67.9	10.00	44.2	0.2	55.8	8.6	12.47	18.88	51.56
OTU 2	96.4	8.9	100	24.30	94.2	4.1	100	18.1	8.17	12.37	63.94
OTU 25	46.4	22.3	42.9	4.71	50.0	3.8	57.7	6.2	6.97	10.55	74.49
OTU 4	89.3	2.4	100	12.80	78.8	0.7	100	11.8	4.57	6.92	81.41
OTU 3	28.6	1.9	46.4	3.01	82.7	8.0	42.3	5.8	4.15	6.29	87.69
OTU 5	85.7	2.9	100	10.60	88.5	2.5	100	9.4	3.62	5.48	93.17
OTU 6	92.9	1.5	100	5.10	71.2	1.1	96.2	2.8	1.60	2.43	95.60
OTU 21	64.3	0.8	85.7	1.57	76.9	1.3	84.6	2.1	0.92	1.39	97.00
OTU 9	32.1	0.0	67.9	0.5	15.4	0.02	71.2	0.6	0.25	0.37	97.37
OTU 7	14.3	0.0	35.7	0.2	65.4	0.6	30.8	0.1	0.24	0.37	97.74
OTU 8	42.9	0.1	75.0	0.6	28.8	0.1	59.6	0.4	0.22	0.33	98.06
OTU 10	46.4	0.2	82.1	0.4	46.2	0.1	67.3	0.4	0.21	0.32	98.38
OTU 18	14.3	0.1	14.3	0.1	46.2	0.4	21.2	0.2	0.19	0.28	98.66
OTU 26	75	0.2	75.0	0.4	15.4	0.03	69.2	0.4	0.18	0.28	98.94
OTU 17	32.1	0.1	46.4	0.1	82.7	0.4	59.6	0.1	0.15	0.23	99.17
OTU 11	17.9	0.0	67.9	0.4	13.5	0.04	53.8	0.3	0.14	0.21	99.39
OTU 27	78.6	0.2	67.9	0.3	0	0	63.5	0.3	0.14	0.21	99.59
OTU 28	82.1	0.2	75.0	0.2	13.5	0.03	67.3	0.1	0.10	0.15	99.74
OTU 22	0	0	7.1	0.01	9.6	0.02	21.2	0.1	0.04	0.06	99.80
OTU 12	7.1	0.1	17.9	0.1	3.8	0.002	7.7	0.008	0.03	0.05	99.85
OTU 13	7.1	0.0	7.1	0.01	7.7	0.04	7.7	0.03	0.03	0.04	99.89
OTU 14	0	0	0	0	23.1	0.07	7.7	0.02	0.02	0.04	99.93
OTU 15	7.1	0.0	10.7	0.02	1.9	0.005	11.5	0.03	0.02	0.03	99.95
OTU 16	0	0	0	0	0	0	1.9	0.04	0.01	0.02	99.97
OTU 23	0	0	0	0	9.6	0.02	1.9	0.001	0.01	0.01	99.99
OTU 19	0	0	3.6	0.03	0	0	1.9	0.004	0.01	0.01	99.99
OTU 20	0	0	0	0	0	0	3.8	0.01	0.003	0.01	100

*Given are the % sample occupancy, % mean relative abundance for each OTU in each habitat, and the average dissimilarity between samples (overall mean = 66.1%). Percentage contribution is the mean contribution divided by the mean dissimilarity across samples, also given is cumulative % contribution. OTUs determined as being core or satellite in a given habitat are highlighted in dark green and light green, respectively*.

Samples were collected in year 4 and 5 of the trial, from continuous OSR, continuous wheat, 1 in 2 OSR, 1 in 3 OSR, virgin OSR (year 4 only) and wheat after OSR (year 5 only). In each case samples were collected from year 4 of the trial in June 2007 (pre harvest), and year 5 of the trial in November 2007 (seedling stage), March 2008 (stem extension) and June 2008 (pre-harvest). Rotations were arranged in a randomized block design, with four replicate plots per rotation, and each plot measuring 24 m × 6 m^2^ (Table S2). At each sampling time, plants were excavated at 6, 12, and 18 m intervals along the length of the plot, with 6 plants collected from each plot. Bulk soil was collected at the same intervals using a 30 cm auger, and pooled within each plot. Plant roots were shaken free of loose soil, all lateral roots were excised from the tap root, and cut into approximately 5 mm sections. The roots and closely adhering soil were designated rhizosphere (for this study explicitly “rhizosphere” includes both root tissue and root-associated soil). Equal amounts of rhizosphere were combined from the 6 plants from within each plot, mixed, and 0.5 g representative sub-samples collected for molecular analysis. Bulk soil was sieved though a 3 mm sieve, taking care to avoid inclusion of roots, and a 0.5 g sub-sample removed for molecular analysis. DNA was extracted from rhizosphere and bulk soil using a FastDNA® SPIN kit for soil (MP Biomedicals LLC, UK) following the manufacturer's guidelines for all steps, except to use a Mini Beadbeater-8 cell disrupter for a 3 min period in place of a FastPrep® machine (Biospec products, Inc., USA). 10 μL of the original DNA was diluted with 40 μL of molecular grade water to give a 1:5 diluted stock solution, which was used for PCR.

### PCR amplification and sequencing of plasmodiophorid 18S rRNA genes

A reference pan-eukaryote alignment (Glücksman et al., 2011) was used to design the new plasmodiophorid-specific primer pair 1301f (5′-GATTGAAGCTCTTTCTTGATCACTTC-3′) and 1,801 g (5′-ACGGAAACCTTGTTACGACTTC-3), which amplify the V7-V9 region of the 18S rRNA gene (18S rDNA). The specificity of these primers was tested by (1) Blastn searches against NCBI GenBank nr/nt database, (2) alignment against the set of plasmodiophorid sequences in Neuhauser et al. (2014), and (3) amplifying two DNA samples separately positive for *Plasmodiophora brassicae* and *Polymyxa graminis*, plus a subset of six soil samples from the present study. Gel electrophoresis showed that all reactions produced a single band of the expected size. The amplicons for *P. brassicae* and *P. graminis* were directly sequenced and yielded single partial 18S rDNA sequences of c. 500 bp long that were 99–100% similar to voucher sequences for those species. Products from the soil samples were cloned and sequenced as described in Neuhauser et al. (2014). Blastn and phylogenetic analyses (see below) confirmed that all 48 successfully sequenced clones grouped within the plasmodiophorid clade.

To generate amplicons for 454 Sequencing, PCR reactions were carried out using 1 μl of DNA (at a final reaction concentration of 0.2 μM) extracted from soil and rhizosphere samples in 24 μl MyTaq HS mastermix (Bioline, London UK). 10-bp MIDs and A and B 454 adaptors were then ligated onto the amplicons, which were cleaned using AMPure XP beads at a ratio of 0.6:1 and samples were equimolar pooled following quantitation using a Shimadzu MultiNA (Milton Keynes, UK). Sequencing was performed on a Roche 454 GS Junior pyrosequencer (454 Life Sciences/Roche Applied Biosystems, Nurley, NJ, USA) at Micropathology Ltd (Coventry, UK) entirely according to the manufacturers protocol with no deviations (libL emPCR kit) (Roche 454 Sequencing system software manual, v 2.5p1). The sequence data are available via NCBI SRA Study number SRP125323.

### Bioinformatic processing of 454 sequence data and phylogenetic analyses

**Figure 1:**
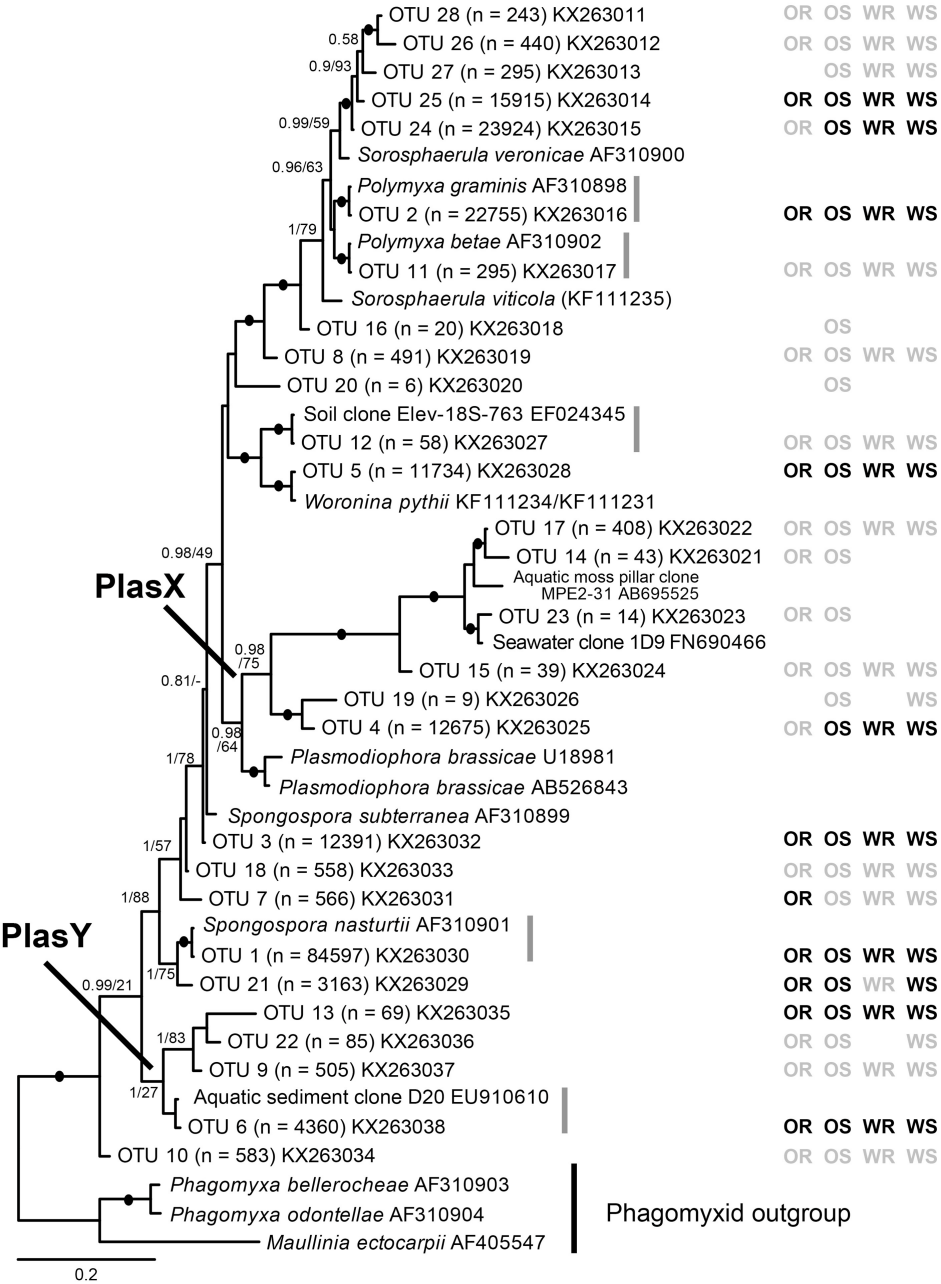
Bayesian analysis of 18S rDNA sequences of 28 plasmodiophorid OTUs generated in this study with all available reference sequences covering the same (V7-V9) 18S region. The matrix to the right of the tree indicates whether the OTUs were detected in oilseed rape rhizosphere/bulk soil (OR/OS) and wheat rhizosphere/bulk soil (WR/WS). Black symbol, core taxa; gray, satellite; absent symbols, not detected. The tree contains 44 taxa; 1625 positions included in the analysis (of which the OTU phylotypes account for c. 450 bp). Support values are shown for Bayesian posterior probabilities >0.75 and Maximum Likelihood (RAxML) bootstrap value >75%, or lower at nodes of particular relevance to this study are mapped onto the Bayesian tree. Black filled circles indicate support of >95% bootstrap and 0.95 posterior probability. Scale bar represents evolutionary distance in changes per site.

QIIME 1.8.0 software (Caporaso et al., 2010) was used to filter the raw sequence files according to a quality score of 25, sequence length between 200 and 1,000 bp, zero primer mismatches, up to six homopolymers, zero ambiguous bases and a maximum of 1.5 barcode errors. The FASTA files were de-multiplexed and partitioned based on sample identifiers. The trimmed sequences were then incorporated into the UPARSE pipeline (Edgar, 2013) to remove singletons (OTUs with < 2 sequences across all samples). The amplicon reads were clustered into OTUs (at 97% sequence similarity). The I-ins-i algorithm in Mafft (Katoh and Standley, 2013) was used to align these with plasmodiophorid sequences from Neuhauser et al. (2014) that included the V7 to V9 18S region amplified by the primers developed for this study. The alignment was then refined by eye. Three OTUs branched robustly within the non-phytomyxid outgroup and were shown to be angiosperm sequences. OTUs that were different from a sequence in the reference database by three or more nucleotide positions in two or more variable regions of the amplicon were considered distinct lineages; those more similar to reference sequences were considered to belong to the reference lineage. OTUs that were distinguished only by nucleotide differences in conserved regions were considered non-distinct and removed, leaving a total of 28 OTUs. These sequences were submitted to GenBank (Accession numbers KX263011- KX263038). The refined alignment for Figure 1 were analyzed in RAxML (Stamatakis, 2006, 2014) BlackBox (GTR model + gamma; all parameters estimated from the data); bootstrap values were mapped onto the highest likelihood tree obtained (Stamatakis et al., 2008). Bayesian consensus trees were constructed using MrBayes v 3.2 (Ronquist et al., 2012) in parallel mode (Altekar et al., 2004) on the Cipres Science Gateway (Miller et al., 2010). Two separate MC^3^ runs with randomly generated starting trees were carried out for 4 million generations each with one cold and three heated chains. The evolutionary model applied included a GTR substitution matrix, a four-category autocorrelated gamma correction and the covarion model. All parameters were estimated from the data. Trees were sampled every 100 generations. One million generations were discarded as “burn-in” (trees sampled before the likelihood plots reached a plateau) and a consensus tree was constructed from the remaining sample.

### Statistical analysis

Phylotypes were partitioned into core and satellite taxa groups as previously described (van der Gast et al., 2011). Fisher's alpha diversity within each sample community was performed using PAST (Paleontological Statistics, version 3.01) program, available from the University of Oslo (http://folk.uio.no/ohammer/past). Fisher's alpha was chosen as it is a measure of diversity that is relatively unaffected by variation in sample size, and completely independent if *N* individuals > 1000 (Magurran, 2004). Two-sample *t*-tests with Bonferroni correction, regression analysis, coefficients of determination (*r*^2^), residuals and significance (*P*) were calculated using XLSTAT (version 2015.1.01, Addinsoft, Paris, France). Bray-Curtis quantitative index of dissimilarity, analysis of similarities (ANOSIM), and similarity of percentages (SIMPER) were performed using the PAST program (Hammer et al., 2001). The Bray-Curtis index was used as the underpinning community dissimilarity measure for both ANOSIM and SIMPER.

## Results

From the 160 samples, 196,196 plasmodiophorid sequences remained after quality screening, with an average sequence read length of 404 bp and an average of 1,226 sequences per sample. These resolved into 28 plasmodiophorid OTUs (plus three discarded plant OTUs), whose phylogenetic position in relation to characterized plasmodiophorids with full 18S rDNA sequence overlap with the OTU amplicons is shown in Figure 1. Gray vertical lines to the right of the branch tip labels indicate the five OTUs that were effectively identical (allowing for low levels of PCR/sequencing error) to sequences in public databases. The other 22 OTUs were all novel, although some are likely to correspond with phylotypes shown in Neuhauser et al. (2014), which can't be directly tested as the amplicon regions do not overlap and therefore preclude phylogenetic comparison. Three OTUs (2, 11, 1) were identical to named plasmodiophorids: *Polymyxa graminis, P. betae*, and *Spongospora nasturtii* respectively. The OTUs were distributed across the known phylogenetic range of plasmodiophorids. Ten OTUs were closely related to characterized plasmodiophorids (Neuhauser et al., 2014): *Sorosphaerula veronicae* (associated with the herb *Veronica spp*.), *S. viticola* (vitaceous vines), *Polymyxa graminis* (Poaceae) and *P. betae* (Chenopodiaceae/Ameranthaceae), *Woronina pythii* (parasite of the oomycete *Pythium spp*.), *Spongospora subterranea* (Solanaceae), and *S. nasturtii* (watercress). The other OTUs were not clearly related to known plamodiophorids, and in some cases (OTUs 17, 14, 23, 15, 19, 4, and OTUs 13, 22, 9, 6) formed two diverse clades whose only previously known members were from environmental sequencing studies. The first of these clades is particularly interesting as it has a moderately well supported sister relationship with the clubroot pathogen *Plasmodiophora brassicae*, with OTUs 4 and 19 the most similar to *P*. *brassicae*, but OTUs 14, 15, 17, and 23 are more closely related to sequences from the Baltic Sea (FN690466) and a freshwater lake moss pillar in the Antarctic (AB695525), both high latitude habitats. Furthermore, this clade is apparently absent from the environmental survey in Neuhauser et al. (2014); we hereon refer to this clade as PlasX. The second clade includes the freshwater-derived EU910610 and two previously detected environmental sequences from the Volga floodplain (Neuhauser et al., 2014); which we refer to subsequently as the “PlasY” clade.

**Figure 2:**
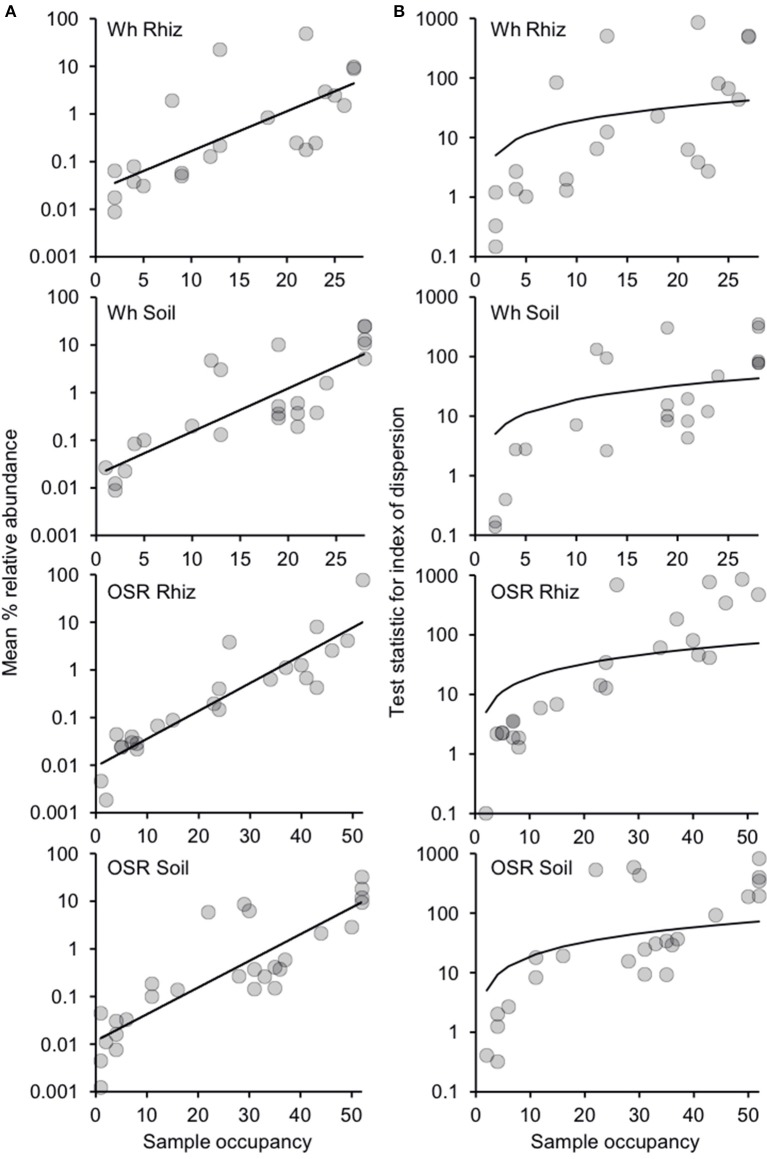
Distribution and dispersal of plasmodiophorid OTUs among the rhizosphere and soil habitats. **(A)** Distribution-abundance relationships for each habitat, depicted as the number of samples for which each detected OTU was observed, plotted against the mean % relative sequence abundance (log_10_ scale) of that OTU among all samples within each habitat (Wheat Rhizosphere, *r*^2^ = 0.53, *F*_(1,20)_ = 22.6, *P* < 0.0001; Wheat Soil *r*^2^ = 0.68, *F*_(1,22)_ = 47.4, *P* < 0.0001; Oil Seed Rape Rhizosphere, *r*^2^ = 0.84, *F*_(1,22)_ = 112.5, *P* < 0.0001; Oil Seed Rape Soil, *r*^2^ = 0.74, *F*_(1,26)_ = 75.1, *P* < 0.0001). **(B)** Random and non-random dispersal of plasmodiophorid OTUs through each habitat. Visualized by decomposing the overall distribution using an index of dispersion based on the ratio of variance to the mean abundance for each OTU. This is plotted against the number of samples for which the OTU was present in that community. The line depicts the 2.5% confidence limit for the χ^2^ distribution. Taxa that fall below this line follow a Poisson distribution, and are randomly distributed and are considered satellite taxa, whereas those that are above the line are non-randomly distributed and are considered core taxa. The 97.5% confidence limit was not plotted, as no taxon fell below that line.

Since there were only minor effects of sampling time on community composition (Table S1), plasmodiophorid samples were separated into four distinct habitat types from across the 2007 and 2008 seasons: wheat rhizosphere (*n* = 28), wheat bulk soil (*n* = 28), OSR rhizosphere (*n* = 52), and OSR bulk soil (*n* = 52). Subsequently, the relative abundance and distributions of the plasmodiophorid OTUs were analyzed within a metacommunity framework for each habitat. Distribution abundance relationships (DARs) were plotted to ascertain whether each habitat metacommunity exhibited a significant positive DAR and therefore represented a coherent metacommunity in each instance (Figure 2A). Consistent with this prediction, for each habitat, the abundance of individual OTUs was significantly correlated with the number of sample communities that they occupied. Next, DARs were objectively partitioned into core and satellite OTU groups by decomposing the overall distribution using the ratio of variance to the mean abundance for each OTU. The variance to mean ratio, or index of dispersion, is an index used to model whether taxa follow a Poisson distribution, falling between the 2.5 and 97% confidence limits of the χ^2^ distribution. The indices of dispersion were plotted against sample occupancy for OTUs in each habitat metacommunity (Figure 2B).

Of the 22 OTUs that comprised the wheat rhizosphere metacommunity, eight were non-randomly distributed and classified as core OTU group members. 14 OTUs were randomly distributed across samples, falling below the 2.5% confidence limit line, and were classified as satellite OTUs. The phylogenetic distribution of core and satellite OTUs, and the samples in which they were detected are shown on Figure 1. Of the 24 OTUs in the wheat soil metacommunity, 9 were core and 15 satellite. Within the OSR rhizosphere and OSR soil metacommunities there were 8 and 9 core OTUs and 16 and 19 satellite OTUs, respectively. Further, the core group OTUs accounted for the majority of relative abundance in each habitat: wheat rhizosphere, 97.8%; wheat soil, 96.7%; OSR rhizosphere, 97.8%; and OSR soil, 96.9%.

**Figure 3:**
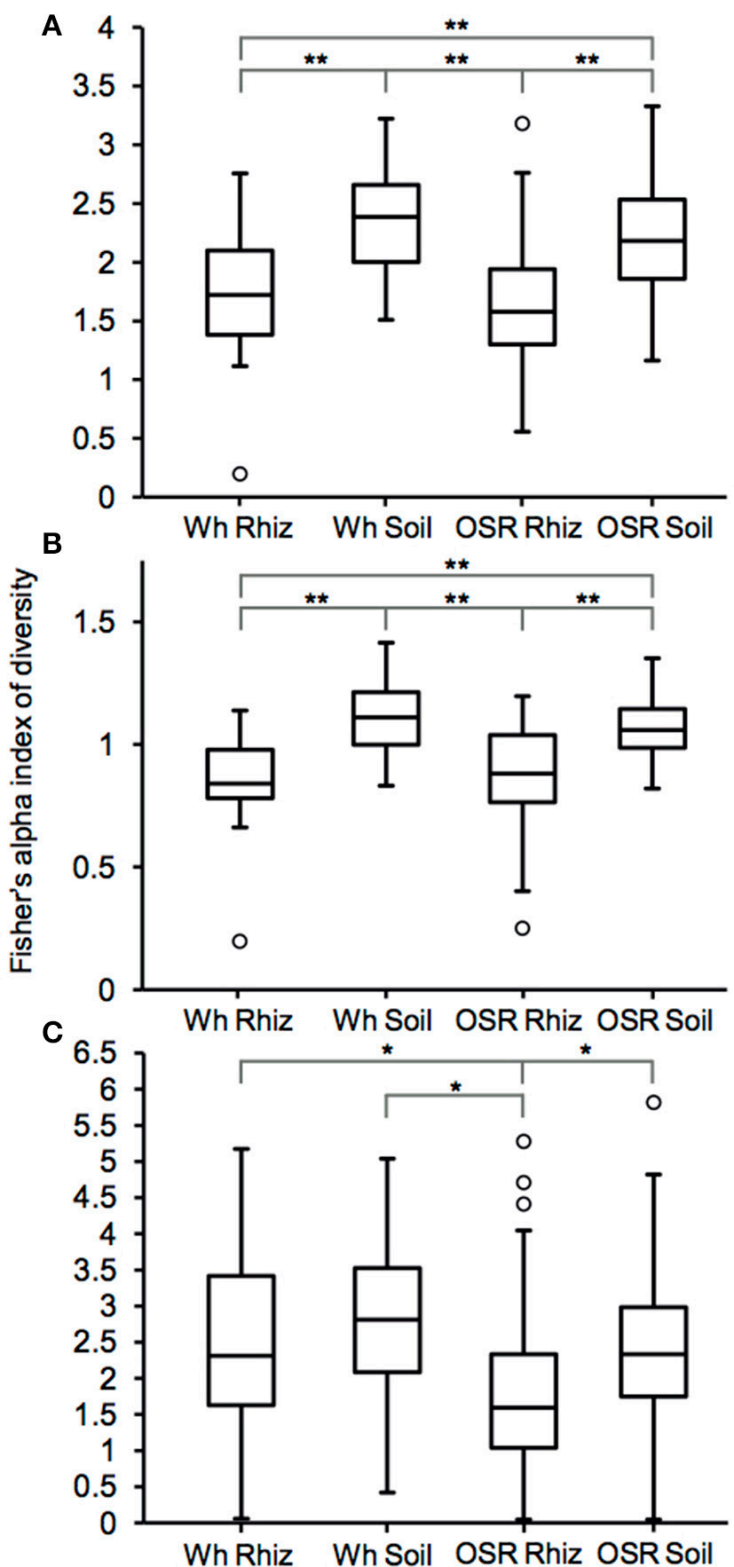
Box plot comparisons of plasmodiophorid diversity between habitats for **(A)** all, **(B)** core, and **(C)** satellite OTUs using Fisher's alpha index of diversity. Boxes represent the interquartile range (IQR) between the first and third quartiles and the line inside represents the median. Whiskers denote the lowest and highest values within 1.5 × IQR from the first and third quartiles, respectively. Circles represent outliers beyond the whiskers. Asterisks denote significant differences in comparisons of diversity at the *P* < 0.05 level determined by two-sample *t*-tests ^*^P < 0.05 and ^**^P < 0.005).

Plasmodiophorid diversity between habitats was compared using Fisher's alpha index of diversity (Figure 3). For the whole metacommunities, mean sample diversity was not significantly different between the wheat and OSR rhizosphere (*P* = 0.28) and wheat and OSR bulk soils (*P* = 0.18), but significantly different at the *P* < 0.05 level in all other instances (Figure 3A). These patterns of diversity were also reflected between core OTU groups: wheat and OSR rhizosphere, *P* = 0.76; and wheat and OSR soil, *P* = 0.14 (Figure 3B). Within the satellite OTU groups, although diversity was more variable, OSR rhizosphere mean diversity was significantly lower when compared to each of the soil satellite groups; *P* > 0.05 in all instances (Figure 3C).

**Figure 4:**
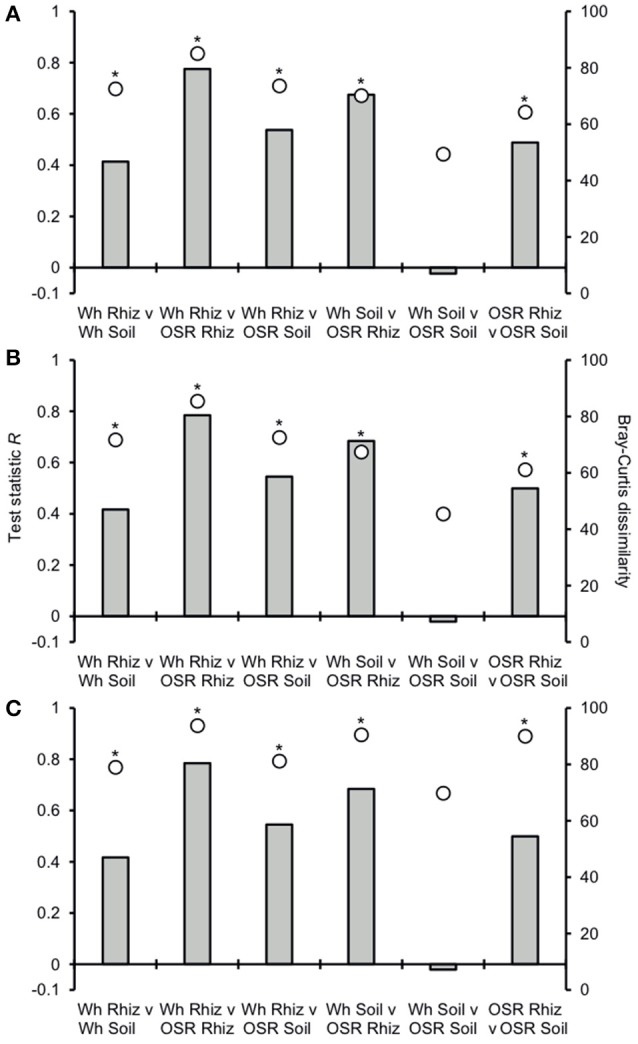
Analysis of similarities (ANOSIM) and community dissimilarity between habitats for **(A)** all, **(B)** core, and **(C)** satellite plasmodiophorid OTUs. Given is the ANOSIM test statistic (*R*, as columns) and probability (*P*, asterisks) that two compared groups are significantly different at the *P* < 0.05 level (all significant differences were less than *P* < 0.0001). ANOSIM *R* and *P* values were generated using the Bray–Curtis measure of dissimilarity. *R* scales from +1 to −1. +1 indicates that all the most similar samples are within the same groups. *R* = 0 occurs if the high and low similarities are perfectly mixed and bear no relationship to the group. A value of −1 indicates that the most similar samples are all outside of the groups. Also given are the Bray-Curtis quantitative measures of dissimilarity between groups, denoted as circles.

**Figure 5:**
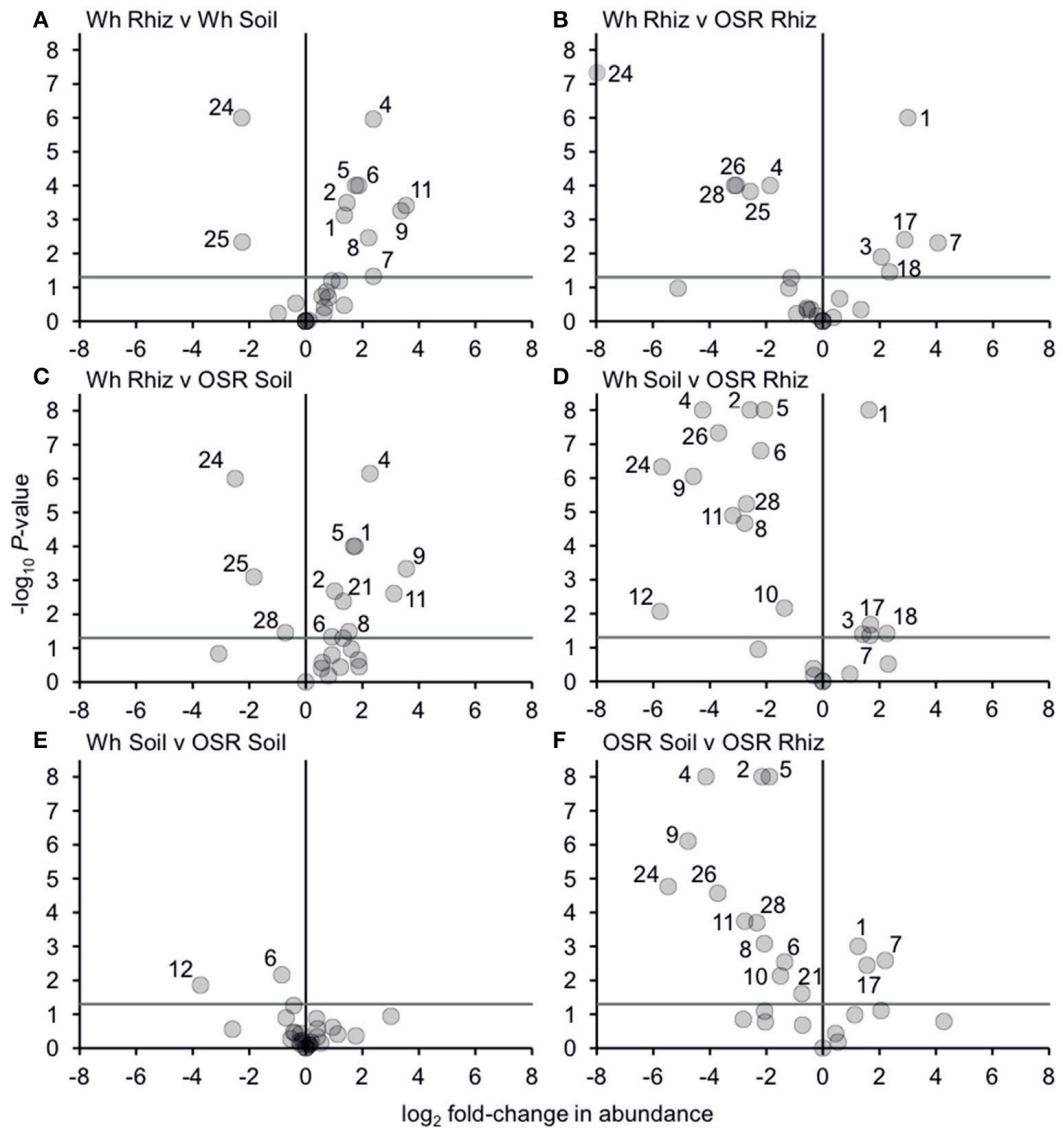
Changes in plasmodiophorid OTU abundances between habitats. Visualized using volcano plots displaying fold-changes in relative abundance of OTUs between compared habitats. Positive and negative values represent increases and decreases in relative mean % OTU abundance within a habitat when compared to another habitat. The gray horizontal line depicts *P* = 0.05, with OTUs above that line having significant fold-changes in relative abundance, whereas those falling below the line are not significant. Numbers for OTUs with significant fold-changes in abundance are also given.

Analysis of similarities (ANOSIM) tests demonstrated that the whole, core, and satellite groups compared between habitats were highly dissimilar and significantly divergent from each other (*P* < 0.0001, in all instances; Figure 4), with the exception of wheat and OSR soil OTU groups which were not significantly different (*P* < 0.05). Similarity of percentages (SIMPER) analysis of the whole metacommunity was used to identify those OTUs that contributed most to the dissimilarity between the four habitat metacommunities. These OTUs are listed in Table 1, with typically, the abundant core OTUs contributing most to the compositional dissimilarity between metacommunities. The impact of habitat type was assessed further at the individual OTU level using volcano plots, by plotting fold-change in relative mean abundance against significance (*P*) values from two-sample *t*-tests of differences in relative abundance for each taxon (Figure 5). Minimal significant impact was observed between wheat and OSR soil and wheat and OSR rhizosphere metacommunities, respectively. While more pronounced significant fold-changes in relative abundance were observed between rhizosphere and soil metacommunities, irrespective of whether planted with wheat or OSR. ANOSIM showed that rotation (i.e., crop frequency over the preceding 3 years) had no effect on the whole rhizosphere plasmodiophorid communities in OSR, but did affect the bulk soil community (Table S1A). However, for wheat there was evidence for effects of rotation on composition of both rhizosphere and bulk soil communities. These effects were mediated through effects on the core community, with no rotational effects on community composition for rhizosphere or bulk soil of either crop in the satellite community (Table S1B,C).

## Discussion

Our group-specific PCR approach coupled with HTS showed that plasmodiophorids are common and diverse in both rhizosphere and bulk agricultural soils. Positive relationships between abundance and distribution have been observed at many spatial scales for taxa when classified into different types of ecological organization (for example, guild or community; Guo et al., 2000). Within the current study, we also observed significant positive DARs (Figure 2A). This indicated that plasmodiophorid OTUs, that were widely distributed throughout each habitat metacommunity were more locally abundant than the taxa with a more restricted distribution. Therefore, as has been observed for other ecological communities, the commonness and rarity of plasmodiophorid taxa within the different metacommunities was found to be related to their occupancy in the local communities (van der Gast et al., 2011), and allowed decomposition of DARs to objectively catergorize core and satellite OTUs (Figure 2B).

Plasmodiophorid assemblage differed strongly between the rhizospheres of OSR and wheat and between all rhizosphere and bulk soil samples. The only non-significant community comparison was between the two bulk soil samples. Therefore the plants clearly exerted a selective force modifying the plasmodiophorid community in rhizosphere/root samples. It is known that the exudates of certain roots trigger germination of resting spores of *P. brassicae* and then can serve as alternative hosts (Friberg et al., 2006; Rashid et al., 2013), and it is likely that root exudates stimulate the germination of other phytomyxid species in a similar way. Plasmodiophorid diversity was lower in the rhizosphere compared to bulk soil, illustrated by significantly lower Fisher's alpha diversity indices, particularly with respect to core OTUs in both wheat and OSR rhizospheres (Figure 3). Further, Plasmodiophorid community composition was significantly different in this respect (Figure 4). In these cases the rhizosphere is selective: recruiting a subset of the pool of diversity from the bulk soil into the rhizosphere microbiota. This trend has been observed in other microbial taxa in the rhizosphere (Morgan et al., 2005). Even if the change of local biodiversity is simply caused by missed detection of some OTUs due to relatively very high representation of others, or if its caused by an imbalance in copy numbers of the genes, it is indicative of a diversity shift between rhizosphere and bulk soil habitats. Significant shifts in the frequency of OTU occurrence in the rhizosphere implies a positive or negative functional relationship between plasmodiophorids and plants, enabling us to infer a functional relationship between an environmental sequence and its hosts potentially offering a tool for the identification of secondary interactions. In some cases these involved characterized plasmodiophorids, which corroborated or extended existing knowledge (Braselton, 2001). However, the majority of the OTUs defined in the study were phylogenetically distant from characterized species.

Bacterial and fungal communities inhabiting the rhizosphere are shaped by a variety of factors, including plant genotype, rotation and plant age (Hilton et al., 2013; Chaparro et al., 2014). In our study, most variation in plasmodiophorid rhizosphere communities was associated with plant species, with rotation having minor effects on the community in wheat but not OSR. In OSR and wheat crops 15 and 11 OTUs respectively were significantly differently distributed between plasmodiophorid metacommunities in rhizosphere compared to soil habitats (Figures 5A,F). However, the community shifts were not the same in each case. In the OSR rhizosphere all but three of the 15 significantly different OTUs decreased in relative mean % abundance compared to OSR soil OTUs (Figure 5F). The three OTUs with increased abundance in the OSR rhizosphere were OTU 1 (the brassicaceae-associated *Spongospora nasturtii*), OTU 7 (*Spongospora*-like), and OTU 17, which groups with the environmental PlasX clade. Of the other 12 OTUs which had decreased abundance in rhizosphere relative to soil, 2, 4, 5, 9, 24, and 26 had the greatest abundance shifts and lowest *P*-values: OTU 2 is the largely cereal-associated *Polymyxa graminis* which will only form secondary, alternative infections in OSR roots, OTU 4 is a novel member of PlasX, more deeply-branching than OTU 17, OTU 5 is the oomycete parasite *Woronina pythii*, OTU 9 groups in the PlasY clade, and OTU 24 and 26 are related to the Plantaginaceae-associated *Sorosphaerula veronicae* (Figure 1). These may be associated with other angiosperm species (e.g., weeds; *Veronica* spp. are abundant agricultural weeds) present in the bulk soil area but not in the crop plant rhizospheres, or other organisms (e.g. oomycetes) associated with other plant species.

The sequence of OTU 1 was very similar to that of *Spongospora nasturtii* (AF310901), differing only in one homopolymer region in the amplicon, and therefore possibly representing the same sequence in this region. However, the traditional taxonomic concept of phytomyxids based on the morphology and arrangement of the resting spores is not always well reflected in molecular phylogenies. Different plasmodophorid species can have very similar 18S rDNA sequences (Neuhauser et al., 2014), so this does not prove that OTU 1 is *S. nasturtii*, but it is certainly very closely related. *S. nasturtii* causes crooked root disease of water cress (*Nasturtium officinale*, a brassica) in the UK (Claxton et al., 1998), and also Belgium, France, and the US (CABI, EPPO, 2011). Therefore we now show that this or a closely related lineage is preferentially associated with OSR (another brassica), and also negatively associated with wheat rhizosphere relative to proximal bulk soil. Overall the increase of OTU1 in the OSR rhizosphere is interesting from a biological point of view as it indicates a wider host range within brassicas including OSR of the crook root parasite *S. narsturtii* or the existence of a closely related lineage capable of infecting OSR.

Equally interesting are OTUs representing currently uncharacterized lineages detected only via our environmental sequencing, which were also responding positively and negatively to the rhizosphere habitat, indicating that these lineages interact with the respective plant or another closely plant-associated organism. It is notable that in addition to OTU 7, which has no close relative in Figure 1 and OTU 17 in the PlasX clade, the related OTUs 14 and 23 were also positively associated with OSR, being detected only with OSR in this study, in both soil and rhizosphere. Whether or not these interactions are those of a fully compatible host-parasite pathosystem or if they are of an “alternative host” type cannot be answered at this stage. But any form of increased interaction of host and parasite will have an ecological role which quickly can translate into productivity changes in the agricultural context.

The wheat rhizosphere showed different plasmodiophorid associations compared to bulk soil from OSR plots. Of the 11 OTUs which were significantly differently distributed between wheat rhizosphere and proximal bulk soil, only two were relatively more abundant in the rhizosphere: OTU 24 (which decreased from OSR soil to rhizosphere), and the closely related OTU 25, both of which are closely related to *Sorosphaerula veronicae* but almost certainly not the same species. Molecular phylogenies have shown previously that *Sorosphaerula, Polymyxa* and *Ligniera* form a well-supported clade with not as well defined borders between the genera (Neuhauser et al., 2014). The known hosts of *S. veronicae* include different *Veronica* spp. (Plantaginaceae). It is worth noting that a species called *S. radicalis* has been described from the root hairs of different grasses from the UK (Ivimey Cook and Schwartz, 1929) as well as *Ligniera pilorum* which was reported from *Poa* spp. root hairs (Karling, 1968). However, no DNA sequences of these species are available It is therefore possible that some of the OTUs found here might correspond to already described species without a validated DNA record.

The nine OTUs less frequently detected in wheat rhizosphere than bulk soil (OTUs 1, 2, 4, 5, 6, 7, 8, 9, 11; Figure 5A) include seven (2, 4, 5, 6, 9, 11) that were also more abundant in OSR bulk soil than OSR rhizosphere. However, the other two (OTUs 1 and 7; *Spongospora* relatives) were more abundant in OSR rhizosphere than soil, converse to the situation in wheat. Directly comparing wheat with OSR rhizosphere samples (Figure 5B), the *Sorophaerula* relatives OTUs 24, 25, 26, 28, and the PlasX member OTU 4 were significantly less abundant in OSR, whereas OTUs 1, 3, 7, 18 (*Spongospora* relatives), and 17 (long-branched PlasX) were significantly more abundant in OSR. Therefore the rhizosphere habitat of both crop types positively and negatively selected plasmodiophorid lineages from the surrounding bulk soil. Although these sets of OTUs overlapped, they were not identical, with different recognized genera being significantly shifted in each case. Further, the direction of OTU abundance shifts differed between crop species. It is important to note that different plasmodiophorid taxa are associated with particular plant hosts with which they from fully compatible interactions. But from all the species mentioned above in relation to wheat and OSR rhizospheres it is known that a number of other hosts can be utilized (at least) for a short time. Our results suggest some strong associations between protist and plants that were not previously recognized, and it will be up to future research to indentify the biological basis of these associations.

Three OTUs were found in soil only and not any rhizosphere samples—OTUs 16 and 20 (both branching basally to the *Sorosphaerula* clade), and OTU 19 (sister to OTU 4, which showed negative abundance shifts in both wheat and OSR rhizospheres relative to soil). These OTUs are not sufficiently phylogenetically close to any characterized plasmodiophorid to infer their ecological roles. It is possible that they are directly excluded by other, positively rhizosphere-associated plasmodiophorids or other organisms. Another possibility is that they may be symbionts of other plant species present at the site in limited abundance (weeds) or that they are present in the form of resting spores, or that these lineages are symbionts of other organisms occurring throughout the soil which are relatively less abundant in the more specialized rhizosphere communities. The only lineages with significantly different distribution between the OSR and wheat bulk soil samples were OTUs 6 and 12, which both group in clades not known to be associated with higher plants: OTU 6 in PlasY (Figure 1), closely related to an environmental sequence from a freshwater aquifer (and therefore possibly a parasite of an aquatic alga or oomycete), and OTU 12 grouping in a clade with *Woronina* (a parasite of oomycetes).

In the light of the high plasmodiophorid diversity detected in this study the absence of any OTU identical or similar to *Plasmodiophora brassicae* is notable. The PCR primers used had no mismatches with available *P. brassicae* sequences. However, there is no history of clubroot disease at the sites studied, so its absence is not unexpected.

We show for the first time that many plasmodiophorids beyond the five well-studied plasmodiophorid agricultural parasites (*P. brassicae, Spongospora subterranea, S. nasturtii, Polymyxa graminis, P. betae*) are present in significant numbers in agricultural soils and in the rhizosphere, even if their known primary hosts are absent or rare. Generally, it is assumed that the distribution of plasmodiophorids follows that of their hosts, but the fact that phytomyxids can use alternative hosts (Neuhauser et al., 2014) means that predicting their diversity and distribution is non-trivial. Within the wheat rhizosphere samples *Polymxya graminis*-like OTUs 2 and 24 were predictably dominant, as wheat is a primary host plant of *P. graminis* (Table 1). On the other hand, the *S. nasturtii*-like OTU 1 dominated the OSR rhizospheres. The primary host of *S. nasturtii* is another brassicaceous plant (*Nasturtium* spp.), therefore the organism represented by OTU1 may interact in a similarly compatible way with a range of brassicas. *Spongospora* sp. are known vectors for plant viruses (Merz and Falloon, 2009), pointing to an additional interesting aspect of this interaction.

The DNA-based detection used in this study does not itself discriminate between active and dormant forms. However, in this system it is apparently sensitive enough to strongly indicate shifts in interaction dynamics between host and symbionts. We show that functional as well as phylogenetic and distribution information can be inferred from environmental sequencing (eDNA) methods combined with a structured and biologically informed sampling strategy. Our results show that diverse plasmodiophorid lineages were positively associated with rhizosphere/root samples compared to bulk soil, and that the enriched lineages were different in wheat and OSR rhizosphere/roots indicating that selection processes in the rhizosphere/root play a role in the establishment and persistence of plant-associated phytomyxids with the potential to increase or decrease the load of pathogenic species.

## Data accessibility

DNA sequences: GenBank accessions KX263011- KX263038.

## Author contributons

DB, ST, and GB designed the research, ST and SH performed the research, DB, CvdG, and GB performed the analyses, DB, CvdG, SN, and GB wrote the paper.

### Conflict of interest statement

The authors declare that the research was conducted in the absence of any commercial or financial relationships that could be construed as a potential conflict of interest.
