# Supplementary material for: Plant Rhizosphere Selection of Plasmodiophorid Lineages from Bulk Soil: The Importance of “Hidden” Diversity
Source: Front Microbiol. 2018 Feb 13;9:168. doi: 10.3389/fmicb.2018.00168 (PMC5825890; doi:10.3389/fmicb.2018.00168)
Supplement: Supplementary file 1 [file DataSheet1.pdf]

Table S1 Analysis of similarities (ANOSIM) between habitat communities by crop rotation for (a) whole, (b) core, and (c) satellite plasmodiophorid OTUs. Each Table is split into upper and lower parts, with the lower part showing the ANOSIM test statistic (R) between each rotation, and the upper part giving the probability that the two rotations compared are significantly different ; green highlighted comparisons are significantly different at the level stated. 4 year rotation shows order of crop sequence between 2005 and 2008 (left to right): O, oilseed rape; w, wheat; BS, bulk soil; Rhiz, rhizosphere

a

4 year Rotation	Metacommunity	OOOO Rhiz O	OOOO BS O	OWWO Rhiz O	OWWO BS O	WOWO Rhiz O	WOWO BS O	WWWO Rhiz O	WWWO BS O	OWWW Rhiz W	OWWW BS W	WWOW Rhiz W	WWOW BS W	WWWW Rhiz W	WWWW BS W
**OOOO**	**Rhiz O**		0.0002	0.6594	0.0001	0.5967	0.0001	0.6479	0.0104	0.0004	0.0002	0.0001	0.0001	0.0001	0.0001
**OOOO**	**BS O**	0.463		0.0001	0.0214	0.0003	0.0473	0.024	0.3655	0.0001	0.0072	0.0001	0.1978	0.0001	0.0007
**OWWO**	**Rhiz O**	-0.015	0.437		0.0001	0.6749	0.0001	0.4847	0.0224	0.0004	0.0003	0.0001	0.0001	0.0001	0.0001
**OWWO**	**BS O**	0.703	0.108	0.615		0.0001	0.7534	0.0002	0.3158	0.0005	0.5023	0.0001	0.201	0.0003	0.5623
**WOWO**	**Rhiz O**	-0.013	0.332	-0.017	0.555		0.0003	0.7676	0.0952	0.0002	0.0004	0.0001	0.0002	0.0001	0.0001
**WOWO**	**BS O**	0.572	0.076	0.493	-0.031	0.430		0.0003	0.7708	0.0003	0.699	0.0002	0.4278	0.0008	0.3813
**WWWO**	**Rhiz O**	-0.106	0.362	-0.009	0.638	-0.153	0.476		0.0525	0.0299	0.029	0.0011	0.0013	0.0008	0.0004
**WWWO**	**BS O**	0.524	0.042	0.445	0.054	0.200	-0.109	0.573		0.0285	0.028	0.0182	0.0989	0.0021	0.0182
**OWWW**	**Rhiz W**	0.992	0.987	0.919	0.866	0.822	0.754	1	0.917		0.0274	0.0007	0.0006	0.0021	0.0006
**OWWW**	**BS W**	0.949	0.463	0.810	-0.016	0.766	-0.087	1	0.427	1		0.0012	0.0007	0.0082	0.4271
**WWOW**	**Rhiz W**	0.727	0.589	0.647	0.443	0.591	0.413	0.461	0.328	0.508	0.411		0.0007	0.3535	0.0002
**WWOW**	**BS W**	0.663	0.041	0.585	0.040	0.436	-0.001	0.666	0.193	1	0.724	0.474		0.0001	0.0012
**WWWW**	**Rhiz W**	0.839	0.650	0.785	0.414	0.774	0.394	0.640	0.383	0.417	0.310	-0.005	0.544		0.0007
**WWWW**	**BS W**	0.876	0.294	0.748	-0.017	0.698	0.008	0.929	0.380	1	0.013	0.564	0.281	0.436	

b

4 year Rotation	Metacommunity	OOOO Rhiz O	OOOO BS O	OWWO Rhiz O	OWWO BS O	WOWO Rhiz O	WOWO BS O	WWWO Rhiz O	WWWO BS O	OWWW Rhiz W	OWWW BS W	WWOW Rhiz W	WWOW BS W	WWWW Rhiz W	WWWW BS W
**OOOO**	**Rhiz O**		0.0001	0.6431	0.0001	0.6223	0.0001	0.6523	0.0115	0.0002	0.0005	0.0001	0.0001	0.0001	0.0001
**OOOO**	**BS O**	0.472		0.0001	0.0218	0.0001	0.0516	0.0182	0.346	0.0002	0.0061	0.0001	0.1916	0.0001	0.0004
**OWWO**	**Rhiz O**	-0.014	0.448		0.0001	0.6395	0.0001	0.502	0.0188	0.0002	0.0004	0.0001	0.0001	0.0001	0.0001
**OWWO**	**BS O**	0.709	0.108	0.627		0.0001	0.7715	0.0003	0.324	0.0005	0.4596	0.0002	0.1957	0.0002	0.5344
**WOWO**	**Rhiz O**	-0.014	0.345	-0.015	0.567		0.0001	0.7747	0.0868	0.0002	0.0003	0.0001	0.0003	0.0001	0.0001
**WOWO**	**BS O**	0.579	0.074	0.503	-0.032	0.442		0.0001	0.7652	0.0003	0.671	0.0001	0.4217	0.0002	0.3864
**WWWO**	**Rhiz O**	-0.104	0.372	-0.012	0.642	-0.156	0.477		0.0589	0.0264	0.0302	0.0005	0.0005	0.0006	0.0006
**WWWO**	**BS O**	0.532	0.045	0.449	0.056	0.213	-0.109	0.583		0.0309	0.0261	0.014	0.0988	0.002	0.0159
**OWWW**	**Rhiz W**	0.995	0.990	0.924	0.877	0.833	0.767	1	0.917		0.0283	0.0009	0.0008	0.0024	0.0011
**OWWW**	**BS W**	0.951	0.464	0.821	-0.002	0.777	-0.080	1	0.427	1		0.0009	0.0004	0.0062	0.4113
**WWOW**	**Rhiz W**	0.732	0.598	0.658	0.448	0.608	0.419	0.475	0.340	0.516	0.423		0.0005	0.3764	0.0001
**WWOW**	**BS W**	0.674	0.042	0.596	0.043	0.451	0	0.679	0.200	1	0.730	0.478		0.0002	0.0015
**WWWW**	**Rhiz W**	0.843	0.656	0.797	0.419	0.785	0.398	0.649	0.394	0.414	0.319	-0.007	0.548		0.0007
**WWWW**	**BS W**	0.882	0.295	0.759	-0.013	0.714	0.006	0.931	0.381	1	0.021	0.568	0.281	0.434	

c

4 year Rotation	Metacommunity	OOOO Rhiz O	OOOO BS O	OWWO Rhiz O	OWWO BS O	WOWO Rhiz O	WOWO BS O	WWWO Rhiz O	WWWO BS O	OWWW Rhiz W	OWWW BS W	WWOW Rhiz W	WWOW BS W	WWWW Rhiz W	WWWW BS W
**OOOO**	**Rhiz O**		0.0001	0.6998	0.0001	0.4571	0.0001	0.8087	0.1158	0.0006	0.0018	0.0001	0.0001	0.0001	0.0001
**OOOO**	**BS O**	0.537		0.0001	0.487	0.0001	0.0869	0.0003	0.6739	0.0139	0.8638	0.0012	0.8951	0.0008	0.6046
**OWWO**	**Rhiz O**	-0.023	0.642		0.0001	0.9894	0.0001	0.3554	0.0199	0.0004	0.0001	0.0001	0.0001	0.0001	0.0001
**OWWO**	**BS O**	0.543	-0.005	0.650		0.0001	0.1935	0.0003	0.331	0.0127	0.8761	0.0001	0.7327	0.0006	0.7194
**WOWO**	**Rhiz O**	-0.003	0.539	-0.066	0.547		0.0001	0.2417	0.0141	0.0003	0.0003	0.0001	0.0001	0.0001	0.0001
**WOWO**	**BS O**	0.600	0.053	0.680	0.030	0.578		0.0004	0.2299	0.0014	0.8213	0.0001	0.5159	0.0001	0.4813
**WWWO**	**Rhiz O**	-0.155	0.664	0.035	0.624	0.075	0.852		0.0304	0.0311	0.0288	0.0002	0.0006	0.0016	0.0006
**WWWO**	**BS O**	0.207	-0.073	0.337	0.055	0.234	0.110	0.833		0.0282	0.1148	0.0419	0.5188	0.1451	0.428
**OWWW**	**Rhiz W**	0.687	0.326	0.783	0.398	0.679	0.493	1	0.760		0.0264	0.6786	0.0066	0.6698	0.002
**OWWW**	**BS W**	0.564	-0.174	0.667	-0.192	0.473	-0.152	1	0.323	0.854		0.0533	0.5126	0.4247	0.9112
**WWOW**	**Rhiz W**	0.595	0.232	0.649	0.334	0.599	0.431	0.767	0.256	-0.069	0.238		0.0002	0.568	0.0001
**WWOW**	**BS W**	0.534	-0.063	0.628	-0.036	0.514	-0.009	0.783	-0.023	0.494	-0.024	0.309		0.0004	0.7188
**WWWW**	**Rhiz W**	0.702	0.237	0.752	0.252	0.654	0.360	0.729	0.187	-0.106	-0.001	-0.019	0.239		0.0001
**WWWW**	**BS W**	0.590	-0.020	0.703	-0.034	0.564	-0.004	0.876	0.014	0.479	-0.203	0.430	-0.031	0.323	

Table S2 Cropping history of rotations sampled. Rhisosphere and bulk soil samples were collected from rotations shaded grey, in year 4 (June 2007) and 5 (November 2007, March 2008 and June 2008) of the trial

**Rotation**	**Year of Trial**				
	1	2	3	4	5
Continuous OSR	O	O	O	O	O
Continuous wheat	O	W	W	W	W
OSR 1 in 2	W	O	W	O	W
OSR 1 in 2	O	W	O	W	O
OSR 1 in 3	W	O	W	W	O
OSR 1 in 3	O	W	W	O	W
Virgin OSR/ Wheat after OSR	W	W	W	O	W

(O = OSR, W = wheat)
